# Left ventricular long axis tissue Doppler systolic velocity is independently related to heart rate and body size

**DOI:** 10.1371/journal.pone.0173383

**Published:** 2017-03-13

**Authors:** Roger E. Peverill, Bon Chou, Lesley Donelan

**Affiliations:** Monash Cardiovascular Research Centre, MonashHeart and Department of Medicine (School of Clinical Sciences at Monash Medical Centre), Monash University and Monash Health, Clayton, Victoria, Australia; University of Minnesota, UNITED STATES

## Abstract

**Background:**

The physiological factors which affect left ventricular (LV) long-axis function are not fully defined. We investigated the relationships of resting heart rate and body size with the peak velocities and amplitudes of LV systolic and early diastolic long axis motion, and also with long-axis contraction duration.

**Methods:**

Two groups of adults free of cardiac disease underwent pulsed-wave tissue Doppler imaging at the septal and lateral mitral annular borders. Group 1 (n = 77) were healthy subjects <50 years of age and Group 2 (n = 65) were subjects between 40–80 years of age referred for stress echocardiography. Systolic excursion (SExc), duration (SDur) and peak velocity (s') and early diastolic excursion (EDExc) and peak velocity (e') were measured.

**Results:**

SExc was not correlated with heart rate, height or body surface area (BSA) for either LV wall in either group, but SDur was inversely correlated with heart rate for both walls and both groups, and after adjustment for heart rate, males in both groups had a shorter septal SDur. Septal and lateral s` were independently and positively correlated with SExc, heart rate and height in both groups, independent of sex and age. There were no correlations of heart rate, height or BSA with either e` or EDExc for either wall in either group.

**Conclusion:**

Heart rate and height independently modify the relationship between s` and SExc, but neither are related to EDExc or e`. These findings suggest that s` and SExc cannot be used interchangeably for the assessment of LV long-axis contraction.

## Introduction

The longitudinally oriented myocardial fibres of the left ventricle provide substantial contributions to both left ventricular (LV) ejection and filling [1–3], and echocardiographic measurements which reflect the action of these fibres on mitral annular motion have proven to be clinically useful [4]. Both mitral annular plane systolic excursion and the peak velocity of systolic mitral annular motion (s`) can be used as predictors of a low ejection fraction [5–11], both have also been utilised to demonstrate impairment of long axis systolic function in patients with heart failure and a preserved ejection fraction [12–16], and s` provides prognostic information in heart failure with reduced ejection fraction [17]. The peak velocity of early diastolic mitral annular motion (e`) is now in routine use to assess LV relaxation [18], as a correction factor for E (E/e`) in the estimation of mean left atrial pressure [18], and e` also provides information about prognosis in heart disease [17–23]. Given that an inverse correlation of e` with age has been consistently demonstrated in multiple studies [24–28], it is now well accepted that interpretation of e` requires consideration of subject age. There is also evidence of an inverse correlation between age and LV s`, although this relationship is relatively weak in contrast to the correlation of age with e` [24,27–31], and there are studies which do not support such a relationship [32–35]. Whether the interpretation of measures of LV long axis function should also involve consideration of factors other than age has received little attention.

Resting heart rate varies over a moderate range in normal individuals, is often elevated in association with cardiac pathology, and is an important independent determinant of prognosis in both the general population and those with cardiac disease [36]. In healthy subjects at rest there is an inverse linear correlation of heart rate with the electromechanical duration, as reflected in the QT interval, the interval between the Q wave and aortic valve closure (QS_2_) [37], and the LV ejection time [38]. A positive correlation between the frequency and velocity of myocardial contraction has also been reported [39,40] and therefore a higher resting heart rate might be expected to be associated with both a shorter duration of long axis contraction and a faster contraction velocity. However, findings have not been consistent in the studies which have examined the relationship of heart rate with s` or its M-mode equivalent [7,30,33,41–47]. There are also reasons to consider a relationship of heart rate with e` give that frequency-dependent acceleration of LV relaxation has been demonstrated during pacing mediated increases in heart rate in humans [48], and there have been reports that increases in heart rate have an effect on diastolic LV long axis motion [42,49,50].

Body size could be related to long axis LV function given that there have been reports of a positive correlation of s` with height and body surface area (BSA) in healthy adults [42,46]. On the other hand, e` does not appear to be related to height, but an inverse correlation of e` with weight and body mass index (BMI) has been reported [46,51,52]. As sex is an important determinant of both body size and the duration of electromechanical systole, with QS_2_ being shorter in males [37], an important limitation of previous studies of long axis function is that sex was not always taken into account. Moreover, while it has been suggested that measurement of tissue Doppler velocities alone can only provide only a limited perspective on long axis function [2,53], previous investigations of the effects of heart rate and body size on long axis LV function have generally not included simultaneous examination of the amplitudes of annular excursion, the peak annular velocities and contraction duration. Accordingly, in this study we have investigated the relationships of resting heart rate and body size with long axis LV contraction duration and with both the amplitudes and peak velocities of systolic and early diastolic motion, at the same time also considering possible contributions of age and sex. There were two study groups comprised of individuals free of cardiovascular disease and these have been analysed separately and evaluated for consistency of the findings: healthy adult subjects <50 years of age without hypertension or obesity (Group 1) and adult subjects between 40 and 80 years old who were referred for stress echocardiography, but had a normal LV ejection fraction (EF) and were free of valvular and ischemic heart disease (Group 2).

## Methods

### Subjects

The study design was approved by the Monash Health Human Research and Ethics committee and all clinical investigation was conducted according to the principles expressed in the Declaration of Helsinki. Written informed consent was obtained prospectively from Group 1 subjects, but as Group 2 subjects were identified retrospectively the requirement for consent was waived. Height and weight were measured immediately prior to the echocardiographic study, BSA was calculated using the formula: 0.0001 x 71.84 x (weight [kg])^0.425^ x (height [cm])^0.725^ and BMI was calculated as weight in kilogram per square metre in height (kg/m^2^). Blood pressure was measured during the echocardiographic study with the patient in a supine position. Study group 1 comprised 77 healthy adult subjects <50 years of age. Subjects were eligible if they had no history of cardiac disease, diabetes or hypertension, were on no medication, had a BMI < 30 kg/m^2^ and had a systolic blood pressure (BP) ≤140 and a diastolic BP ≤90 mm Hg at the time of the study. All subjects also had normal LVEF (≥55%) and no more than mild valvular disease as assessed by echocardiography. Study group 2 comprised 65 adult subjects selected from a consecutive population referred for stress echocardiography to investigate for possible ischaemic heart disease. Exclusion criteria included diabetes, renal failure, morbid obesity (BMI > 40 kg/m^2^), preceding beta blocker medication (even if ceased for the study), known myocardial infarction or symptomatic ischaemic heart disease, a LVEF <55%, a regional wall motion abnormality, more than mild valvular disease or a positive test for inducible ischemia.

### Echocardiography

**Fig 1:**
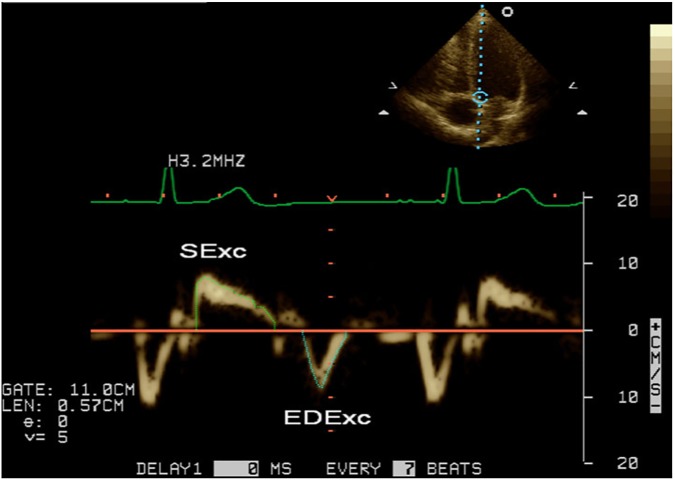
Example of the method for tracing the velocity time integrals of the systolic and early diastolic tissue Doppler signals from the mitral annulus.

Echocardiography was performed on a Sonos 5500 machine (Philips, Amsterdam, The Netherlands) in Group 1 and a Vivid 7 machine (GE Healthcare, Chicago, IL, USA) in Group 2. Studies were stored digitally and measured offline using Xcelera V1.2 L4 SP2 (Philips, Amsterdam, The Netherlands) by one of 2 investigators (LD or BC). Four- and two-chamber 2-dimensional loops of left ventricular contraction were recorded in both groups and used for measurement of LV end-diastolic volume (LVEDV), end-systolic volume and the calculation of the ejection fraction (LVEF) using the biplane method of discs. The length of the LV at end-diastole (LVEDL) from the plane of the mitral annulus to the apical endocardium in the 4- and 2-chamber views was recorded during the measurement of the LVEDV, and the longest dimension from these 2 views has been used [54]. Pulsed-wave TDI was performed in the apical 4-chamber view in both groups and TDI signals of longitudinal mitral annular motion were recorded during non-forced end-expiration apnoea at both septal and lateral borders of the mitral annulus after optimising parallel alignment of the ultrasound beam [46]. Measurements were made of the duration of the systolic (SDur) signal and the time interval between the end of the systolic signal and the commencement of the early diastolic signal as a TDI long axis equivalent of the isovolumic relaxation time (IVRT`). Measurements were made from the systolic signals of the peak velocity (s') and the velocity time integral (systolic excursion; SExc) and from the early diastolic signals of the peak velocity (e') and the velocity time integral (early diastolic excursion, EDExc) (Fig 1). The heart rate was calculated from the R-R intervals of the relevant TDI signals. In Group 1 results from 3 consecutive cardiac cycles were averaged, and in Group 2 measurements were performed on a single cardiac cycle, with evidence of a preceding sinus beat.

### Statistical analysis

Statistical analysis was performed using Systat V13 (Systat Software, Chicago, IL, USA). Continuous variables are presented as mean ± SD. Univariate linear regression analysis was performed to assess the relationships of age, heart rate and measures of body size with TDI variables. Multivariate regression analysis was performed with selected variables to assess the independence and extent of contributions to the observed relationships. Sex was included in multivariate models as a dummy variable (Male = 1, Female = 0). The partial correlation coefficient (β) value is provided in multivariate analyses. The coefficient of determination has been adjusted for the number of terms in the model (adjusted r^2^) and used to estimate the degree of variability in a dependent variable explained by a multivariate model. Apart from decisions regarding inclusion of variables in multivariate models, a p value of <0.05 was considered significant.

## Results

The clinical characteristics and main echocardiographic variables of the two study groups are shown in Table 1. As expected, age, BP and BMI were all higher in Group 2 than Group 1, whereas all the TDI velocities and excursions were lower in Group 2 (p<0.05 for all). Possible confounding influences on the relationships of heart rate, sex and body size with TDI variables were considered in both groups. In neither group were there any differences in heart rate between males and females and nor were there any correlations of heart rate with age, systolic or diastolic BP, height, weight, BSA or BMI (p >0.05 for all). For each group, univariate correlations of TDI variables with age are shown in Table 2, with heart rate are shown in Table 3 and with anthropometric measures are shown in Table 4.

**Table 1:** Clinical and echocardiographic characteristics of the study groups.

	Group 1 (n = 77)	Group 2 (n = 65)
Sex (male: female)	35:42	31:34
Age (years)	31±8 (18–49)	58±10 (42–80)
Height (cm)	170±8 (155–190)	167±10 (148–188)
Weight (kg)	67.4±10.9 (48–98)	76.8±16.1 (46–115)
BSA (m^2^)	1.78±0.18 (1.47–2.22)	1.85±0.22 (1.40–2.36)
BMI (kg/m^2^)	23.2±2.5 (18.4–29.4)	27.6±4.9 (18.3–39.9)
Obesity	0	23 (35%)
Hypertension	0	24 (36%)
Heart rate (bpm)	63±11 (42–92)	70±11 (48–99)
Systolic BP (mmHg)	112±12 (85–138)	124±14 (90–150)
Diastolic BP (mmHg)	67±8 (45–86)	75±11 (40–100)
LVEF (%)	65±5	60±4
LVEDL (cm)	9.1±0.7	8.7±0.6
Septal s` (cm/s)	8.2±1.4	6.6±1.3
Lateral s` (cm/s)	11.9±2.6	7.9±1.9
Septal SExc (cm)	1.4 ± 0.2	1.1 ± 0.2
Lateral SExc (cm)	1.7 ± 0.3	1.2 ± 0.2
Septal e` (cm/s)	10.6±2.3	6.4±1.9
Lateral e` (cm/s)	17.4±3.8	8.4±2.4
Septal EDExc (cm)	0.9 ± 0.2	0.6 ± 0.2
Lateral EDExc (cm)	1.2 ± 0.2	0.7 ± 0.2

**Table 2:** Univariate correlations of age in Group 1 and Group 2.

	Group 1		Group 2	
	r	P	r	P
**Septal SDur**	0.04	NS	0.02	NS
**Septal SExc**	-0.07	NS	-0.33	0.007
**Septal s`**	-0.20	NS	-0.35	0.004
**Septal IVRT`**	0.28	0.015	0.24	0.057
**Septal EDExc**	-0.31	0.006	-0.39	0.001
**Septal e`**	-0.53	<0.001	-0.53	<0.001
**Lateral SDur**	0.05	NS	0.00	NS
**Lateral SExc**	-0.11	NS	-0.33	0.008
**Lateral s`**	-0.08	NS	-0.24	0.05
**Lateral IVRT`**	0.02	NS	0.21	NS
**Lateral EDExc**	-0.45	<0.001	-0.52	<0.001
**Lateral e`**	-0.53	<0.001	-0.60	<0.001

**Table 3:** Univariate correlations of heart rate in Group 1 and Group 2.

	Group 1		Group 2	
	r	P	r	P
**Septal SDur**	-0.59	<0.001	-0.58	<0.001
**Septal SExc**	-0.04	NS	-0.09	NS
**Septal s`**	0.47	<0.001	0.31	0.012
**Septal IVRT`**	-0.27	0.016	-0.28	0.023
**Septal EDExc**	-0.13	NS	-0.14	NS
**Septal e`**	0.07	NS	0.07	NS
**Lateral SDur**	-0.56	<0.001	-0.55	<0.001
**Lateral SExc**	-0.09	NS	-0.06	NS
**Lateral s`**	0.31	0.007	0.12	NS
**Lateral IVRT`**	-0.33	0.003	-0.35	0.004
**Lateral EDExc**	-0.16	NS	-0.07	NS
**Lateral e`**	-0.09	NS	-0.00	NS

**Table 4:** Univariate correlations of tissue Doppler variables with height, weight, body surface area and body mass index.

	Group 1				Group 2			
	Height	Weight	BSA	BMI	Height	Weight	BSA	BMI
**Septal SDur**	-0.34*	-0.33*	-0.35*	-0.19	-0.23	-0.24	-0.27*	-0.13
**Septal SExc**	0.14	0.13	0.14	0.05	0.26*	-0.04	0.08	-0.21
**Septal s`**	0.36*	0.44*	0.44*	0.37*	0.48*	0.14	0.29*	-0.15
**Septal IVRT`**	-0.00	0.15	0.11	0.23*	-0.29*	-0.22	-0.28*	-0.08
**Septal EDExc**	0.04	-0.11	-0.07	-0.21	0.33*	-0.04	0.10	-0.26*
**Septal e`**	-0.09	-0.26*	-0.21	-0.31*	0.23	-0.04	0.05	-0.20
**Lateral SDur**	-0.19	-0.15	-0.17	-0.07	-0.19	-0.41*	-0.38*	-0.37*
**Lateral SExc**	0.09	0.15	0.15	0.19	0.20	-0.00	0.07	-0.13
**Lateral s`**	0.32*	0.33*	0.36*	0.25*	0.39*	0.11	0.23	-0.14
**Lateral IVRT`**	-0.16	-0.26*	-0.23*	-0.23*	-0.03	0.17	0.11	0.18
**Lateral EDExc**	0.08	0.02	0.05	-0.00	0.23	0.06	0.13	-0.07
**Lateral e`**	0.06	-0.03	0.00	-0.08	0.19	-0.02	0.05	-0.13

* p<0.05

### Left ventricular long axis systolic function

#### Group 1

**Fig 2:**
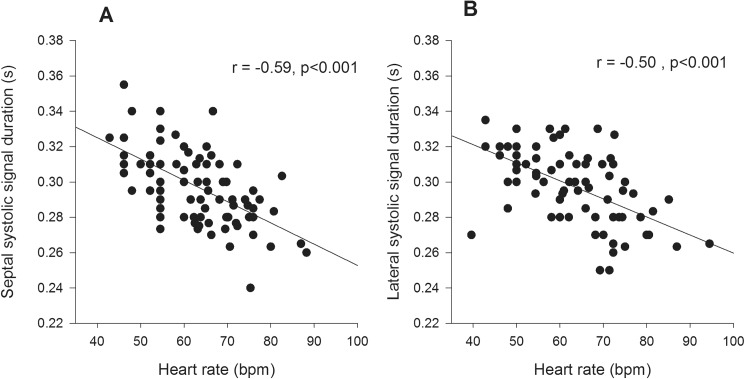
Scatter plots of heart rate v septal systolic signal duration (A) and heart rate v lateral systolic signal duration (B) in Group 1.

**Fig 3:**
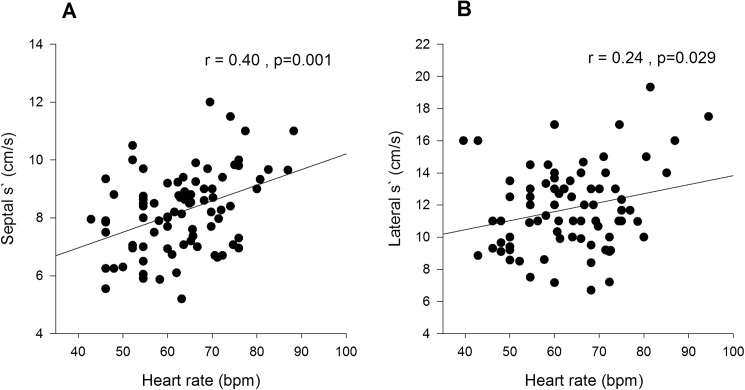
Scatter plots of heart rate v septal s` (A) and heart rate v lateral s` (B) in Group 1.

**Fig 4:**
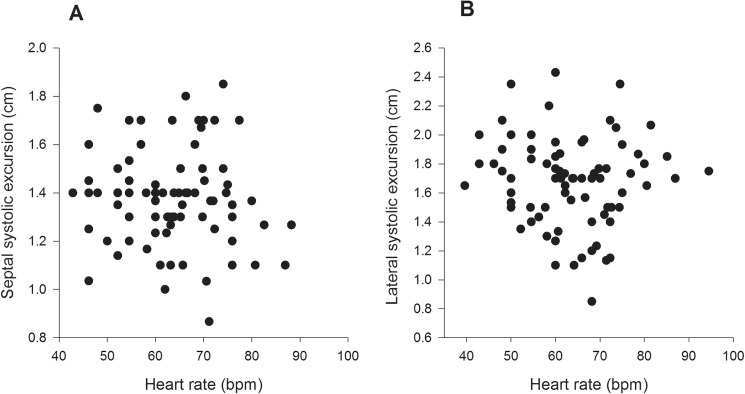
Scatter plots of heart rate v septal SExc (A) and heart rate v lateral SExc (B) in Group 1.

On univariate analysis age was not related to any of the systolic TDI variables (Table 2). Heart rate was inversely correlated with septal and lateral SDur (Fig 2A & 2B), positively correlated with septal and lateral s` (Fig 3A & 3B), but was not correlated with either septal or lateral SExc (Fig 4A & 4B, Table 3). Height, weight and BSA were all inversely correlated with septal SDur, but none of the anthropometric measures were significant correlates of lateral SDur (Table 4). Height, weight and BSA were all positively correlated with both septal and lateral s`, but none of the anthropometric measures were correlated with either septal or lateral SExc (Table 4).

Various multivariate models of SDur are shown in Table 5. Inverse correlations of heart rate with septal and lateral SDur were independent of sex, and male sex was a predictor of shorter septal SDur and a borderline significant predictor of a shorter lateral SDur (p = 0.06). Height became an independent contributor in models of septal and lateral SDur in association with heart rate, but there was no contribution of either height (or BSA) to models of septal and lateral SDur which also included sex and heart rate (p>0.05 for all). SExc was not a correlate of SDur for either the septal or lateral walls in univariate or multivariate analyses (p>0.05 for both). Together heart rate and sex explained 48% and 24% of the variability of septal and lateral SDur, respectively.

**Table 5:** Linear regression analysis of SDur with heart rate, sex and body size in Groups 1 and 2.

		Univariate β	Multivariate β	P value in multivariate model	Cumulative adjusted r^2^
**Group 1**					
**Septal SDur**	Heart rate	-0.59	-0.63	<0.001	0.34
	Male sex	-0.31	-0.38	<0.001	0.48
	Heart rate	-0.59	-0.58	<0.001	0.34
	Height	-0.35	-0.34	<0.001	0.45
	Heart rate	-0.59	-0.57	<0.001	0.34
	BSA	-0.35	-0.33	<0.001	0.44
**Lateral SDur**	Heart rate	-0.48	-0.52	<0.001	0.22
	Male sex	-0.08	-0.19	0.06	0.24
	Heart rate	-0.48	-0.49	<0.001	0.22
	Height	-0.19	-0.22	0.03	0.26
	Heart rate	-0.48	-0.48	<0.001	0.22
	BSA	-0.17	-0.18	0.09	0.24
**Group 2**					
**Septal SDur**	Heart rate	-0.51	-0.52	<0.001	0.25
	Male sex	-0.19	-0.22	0.038	0.37
	Heart rate	-0.51	-0.59	<0.001	0.25
	Height	-0.23	-0.26	0.01	0.38
	Heart rate	-0.51	-0.56	<0.001	0.25
	BSA	-0.27	-0.23	0.026	0.37
**Lateral SDur**	Heart rate	-0.55	-0.56	<0.001	0.29
	Male sex	-0.10	-0.12	0.28	0.30
	Heart rate	-0.55	-0.56	<0.001	0.29
	Height	-0.19	-0.20	0.052	0.33
	Heart rate	-0.55	-0.52	<0.001	0.29
	BSA	-0.38	-0.32	0.002	0.39

Multivariate models of septal and lateral s`were constructed with combinations of the variables SExc, heart rate and either height or BSA. SExc, heart rate and either height or BSA were all independent contributors to the models of septal and lateral s` (Table 6). SExc, heart rate and height together explained 51% and 52% of the variability in septal and lateral s`, respectively. When men and women were analysed separately, SExc, heart rate and either height or BSA were also significant contributors to the model of septal s` (p<0.05 for all) in both sexes, but only SExc and heart rate were significant contributors in the model of lateral s`. LVEDL was also a significant contributor to models of septal and lateral s` which included SExc and heart rate, but addition of LVEDL did not improve the prediction of either septal or lateral s` when either height or BSA were included in the model.

**Table 6:** Linear regression analysis of s` with systolic excursion, heart rate, body size and left ventricular diastolic length.

		Alternative body size options	Univariate β	Multivariate β	P value in multivariate model	Cumulative adjusted r^2^
**Group 1**						
**Septal s`**	SExc		0.47	0.45	<0.001	0.21
	Heart rate		0.47	0.48	<0.001	0.44
		Height	0.36	0.29	0.001	0.51
		BSA	0.44		<0.001	0.56
		LVEDL	0.39		<0.001	0.54
**Lateral s`**	SExc		0.57	0.57	<0.001	0.31
	Heart rate		0.31	0.38	<0.001	0.44
		Height	0.32	0.30	<0.001	0.52
		BSA	0.36		<0.001	0.51
		LVEDL	0.35		<0.001	0.49
**Group 2**						
**Septal s`**	SExc		0.54	0.47	<0.001	0.27
	Heart rate		0.32	0.38	<0.001	0.40
		Height	0.48	0.38	<0.001	0.53
		BSA	0.29		<0.02	0.45
		LVEDL	0.45		<0.001	0.51
**Lateral s`**	SExc		0.71	0.67	<0.001	0.49
	Heart rate		0.16	0.20	<0.02	0.52
		Height	0.39	0.26	0.003	0.58
		BSA	0.24		0.06	0.54
		LVEDL	0.40		0.034	0.55

#### Group 2

On univariate analysis, and in contrast to Group 1, age was inversely correlated with s` and SExc for both the septal and lateral walls, but similar to Group 1, there was no correlation between age and SDur (Table 2). Heart rate was inversely correlated with septal and lateral SDur, was a positive correlate of septal s`but not of lateral s`, and was not related to either septal or lateral SExc (Table 3). Height was a positive correlate of septal and lateral s`and also of septal SExc, but was not correlated with lateral SExc or with septal or lateral SDur (Table 4). BSA was correlated with septal s` but not with septal SExc or with lateral s` or SExc. Weight and BSA were both inversely correlated with septal and lateral SDur (p<0.05 for all).

Various multivariate models of SDur are shown in Table 5. On multivariate analysis including sex and heart rate, heart rate was inversely correlated with septal and lateral SDur, but while male sex was a predictor of a shorter septal SDur, it was not a predictor of lateral SDur (p = 0.28). In combination with heart rate, height was an independent contributor to the model of septal SDur and a borderline significant contributor to lateral SDur (p = 0.052), whereas BSA was a contributor to the models of both septal and lateral SDur (p<0.05 for both). When sex was also included in the model with heart rate, BSA (but not height), remained an independent contributor to the model of lateral SDur (p = 0.003), whereas neither height nor BSA were contributors to the model of septal SDur (p>0.10 for both). Together, combinations of heart rate and sex, heart rate and height or heart rate and BSA could explain 37–38% of the variability of septal SDur, whereas heart rate and BSA could explain 39% of the variability of lateral SDur (Table 5).

Multivariate models of s` were constructed with combinations of the variables SExc, heart rate and height or BSA (Table 6) and similar to Group 1, SExc and heart rate, together with either height or BSA, were all independent contributors to the models of septal and lateral s`. SExc, heart rate and height explained 53% of the variability of septal s` and 58% of the variability of lateral s`. LVEDL was also a significant contributor to models of septal and lateral s` which included SExc and heart rate, but LVEDL did not improve the prediction of septal or lateral s` when either height or BSA were included. There were no contributions of age to models of s` which included SExc, heart rate and height, and sex was not a significant contributor in models of septal or lateral s` when included with either height or BSA (p>0.05 for all).

The group specific equations for the linear regression models of septal and lateral s` which included the variables heart rate and height are shown in Table 7, and calculated values for s` with heart rates of either 55 or 90/min and heights of either 155 or 185 cm are shown in Table 8. The absolute velocities were lower in Group 2 than Group 1, but the percentage differences from the lowest to the highest predicted values were substantial in both groups, e.g. 85% for lateral s` in Group 1 and 89% for lateral s` in Group 2.

**Table 7:** Equations for s` based on heart rate and height in Groups 1 and 2.

**Group 1**	**Septal s`**	0.063 x heart rate + 0.06 x height (cm) - 6.0
	**Lateral s`**	0.081 x heart rate + 0.112 x height (cm) - 12.3
**Group 2**	**Septal s`**	0.044 x heart rate + 0.063 x height (cm) - 7.0
	**Lateral s`**	0.032 x heart rate + 0.073 x height (cm) - 6.6

**Table 8:** Predicted s` in Group 1 and Group 2 based on combinations of two different heart rates and heights.

Heart Rate (/min)	Height	Group 1 septal s` (cm/s)	Group 2 septal s` (cm/s)	Group 1 lateral s` (cm/s)	Groups 2 lateral s` (cm/s)
50	150	6.2	4.6	8.5	6.0
90	150	8.4	6.4	11.8	7.3
50	185	8.3	6.9	12.4	8.5
90	185	10.5	8.7	15.7	9.8

### Left ventricular long axis IVRT` and early diastolic motion

#### Group 1

On univariate analysis age was inversely correlated with e`, EDExc and IVRT`for both septal and lateral walls (Table 2), but there were no correlations of heart rate with septal or lateral EDExc or e` (Table 3), with or without adjustment for age and sex (p>0.05 for all). There were inverse correlations of heart rate with septal and lateral IVRT`, which also remained significant after addition of age to the models (p<0.05 for all). There were no correlations of height, weight or BSA with e` or EDExc at either the septal or lateral walls, but there were inverse correlations of weight and BMI with septal e`.

#### Group 2

On univariate analysis age was inversely correlated with e` and EDExc for both septal and lateral walls (Table 2) but not correlated with IVRT`for either wall. There were no correlations of heart rate with septal or lateral e` or EDExc on univariate analysis, or after adjusting for age or sex (p>0.05 for all). Heart rate was inversely correlated with both septal and lateral IVRT` and the correlations with heart rate remained significant after including age in the models (p<0.05 for all). There was a positive correlation of septal EDExc with height and an inverse correlation of septal EDExc with BMI, but no relation of septal or lateral e` or of lateral EDExc with either height, BSA or BMI (Table 4).

## Discussion

In this cross sectional study of two groups of adult subjects, both with normal LVEF and free of cardiac disease, we have investigated the relationships of resting heart rate, sex, age and body size with the amplitudes of excursion and the peak velocities of both systolic and early diastolic long axis LV motion, and also with the duration of long axis contraction. There were a number of findings which were consistent in both groups and for both the septal and lateral walls: (1) SDur was inversely and independently correlated with heart rate, (2) there was no relationship of SExc with heart rate, height or BSA, (3) s` was only moderately correlated with SExc, and was positively correlated with heart rate and height, independent of SExc, age and sex, (4) IVRT` was inversely and independently correlated with heart rate, and (5) there were no correlations of EDExc or e` with heart rate, height or BSA. The above findings suggest that both resting heart rate and height need to be taken into account when interpreting s` as a measure of LV long axis contraction, whereas, and despite the inverse correlation between heart rate and IVRT`, neither heart rate nor height require consideration for the interpretation of e`.

Our finding of inverse linear correlations between heart rate and LV long axis SDur was not unexpected as inverse correlations of resting heart rate with the duration of the ejection time and LV electromechanical systole have been reported previously in normal subjects with heart rates varying between 40 and 100/min [37,38,55]. Furthermore, an inverse correlation of heart rate with ventricular long axis systolic time intervals has been previously reported in children [56]. However, we believe this to be the first description of an inverse relationship of heart rate with LV long axis contraction duration in adults. More importantly, we believe this to be the first study in which there has been simultaneous assessment of the relationship of heart rate with long axis SDur, SExc and s`, such that the implications of a higher heart rate leading to a shorter duration of contraction on the relationship between SExc and s` has been explored.

Interpretation of the finding of a positive correlation of s` with resting heart rate requires consideration of the Bowditch staircase phenomenon, also known as the treppe effect or the force-frequency relationship [57,58]. While the classic description by Bowditch was of an increase in force of contraction following an increase in heart rate [57], such a relationship has not always been evident in experiments in human myocardium [59,60], whereas a positive association between frequency and velocity of contraction has been a more consistent finding [59–62]. Although peak force may not increase with heart rate, an increase in the peak change in force per unit time (dF/dt) and a decrease in the time to reach peak force could account for an increase in the peak velocity of shortening [60]. Furthermore, while an increase in frequency of contraction can result in an increased peak velocity of shortening, due to the reduced duration of contraction the extent of shortening could remain relatively unchanged [59]. Nevertheless, that the reduction in SDur with increasing heart rate in our study would also be accompanied by an increase in s` could not be assumed as the TDI systolic signal is of a heterogeneous shape, which can vary substantially between individuals [63]. Indeed, a possible reason for the small differences in the relationships of septal and lateral s` could have been differences in the TDI signal shapes between these two walls. Furthermore, while mean contraction velocity must increase if SDur decreases and SExc remains the same, the relationship between heart rate and SExc in our observational study was not predictable as the control of contraction amplitude (and thus SExc) in the intact cardiovascular system is complex, with the amplitude of contraction modified by preload and afterload, as well as by sympathetic drive [58]. For example, a simultaneous increase or decrease in heart rate, SExc and s` is possible during the combined inotropic and chronotropic effects of an increase or decrease in sympathetic drive [64]. It is therefore an important finding that not only was there no relationship between heart rate and SExc in our study groups, but that s` was independently and positively correlated with SExc and heart rate in both groups.

The absence of a relationship between heart rate and SExc does not necessarily exclude an inotropic effect of an increase in heart rate because the amplitude of contraction will also be affected by concomitant changes in loading conditions [64]. Thus, in the absence of augmented metabolic requirements, homeostatic mechanisms maintain cardiac output at a relatively constant level despite large induced changes in the heart rate during atrial pacing in humans [61,65]. A decrease in LV end-diastolic size [65,66], and a reduction in LVEDP [65] have also been shown to occur during increases in heart rate by atrial pacing in humans, presumably due to a concomitant decrease in the volume of venous return per cardiac cycle. Thus, if an increase in heart rate also resulted in a decrease of preload then the amplitude of contraction may not increase even if there is a concomitant inotropic effect.

Despite the lack of a relationship of SExc with either height or BSA in either group, a positive and independent correlation of s`with height (and less convincingly with BSA) was evident in both groups. The lack of any relationship of SExc with body size in our study groups was not expected and there is currently no satisfactory explanation for this observation. The lack of relationship did not appear to be related to a divergence between body size and heart size as LVEDL was positively correlated with height and BSA, and LVEDL was also not related to SExc. Loading conditions also require consideration, but there was no relationship evident between body size and BP or between SExc and BP, and thus no evidence for a positive relationship between body size and afterload as an explanation for the lack of a positive relationship between body size and SExc. With regard to preload, we are not aware of any evidence to suggest a relationship between body size in non-obese subjects and either left atrial pressure or LV end diastolic pressure. On the other hand, it is possible that LV long axis preload may not be directly related to either left atrial pressure or LV end diastolic pressure, particularly if it is considered to be the extent of stretch of the LV wall just prior to contraction. While atrial long axis contraction is an important component of LV long axis stretch [67], the amplitude of mitral excursion due to atrial contraction was also not related to body size in the present study (results not shown).

Height has recognized cardiovascular associations, including variations in heart rate and of the ascending aortic pressure waveform, and these effects also need to be considered when attempting to explain the lack of relationship of body size with SExc and the positive correlation of height with s`. Thus, an inverse relationship between height and heart rate has been described [68,69], but large differences in height are only associated with small absolute differences in average heart rate (< 4/min) [68]. Furthermore, such a relationship cannot provide an explanation for our finding of a positive correlation between height and s` given that no correlation between height and heart rate was evident in the present study. An association of height with the pattern of the ascending aorta pressure waveform is believed to be due to shorter height leading to earlier arrival of reflected waves [69], an explanation supported by the finding of a positive correlation of height with time to the inflexion point of the carotid pressure waveform and an inverse correlation of height with the augmentation index. However, s` peaks well before the inflection point in healthy young subjects [70], and thus the relationship of s` with height in both groups in our study is unlikely to be related to wave reflection. Another consideration is that s` could increase with larger body size if there was an inverse relationship between body size and contraction duration, and there was such a relationship evident on univariate analysis for all but lateral SDur in Group 1.

Sex was considered in our analysis of TDI variables as not only is male sex recognized to be a determinant of a shorter duration of electromechanical systole [37], but sex is also an important determinant of body size. Indeed, male sex was associated with a shorter septal SDur in both groups in the present study and a borderline shorter lateral SDur in Group 1. There was no relationship of male sex with lateral SDur in Group 2, but instead there was an independent inverse correlation of lateral SDur with BSA. In contrast to the relationship with SDur, sex made no independent contribution to the prediction of s` in either group once body size was included in the models. However, given the inherent relationship between sex and body size, it is not possible to exclude contributions of both sex and body size to SDur and s`. It therefore remains possible that male related shortening of contraction duration could be a contributor to the positive correlations of height with s`.

Both s` and mitral annular plane systolic excursion (which is likely to be closely related to SExc) have been used in previous studies to evaluate LV long axis contraction [6,9,12,71,72]. The relationship between s`, and SExc should therefore be of considerable interest, yet it has received little attention in the literature. A notable finding of the present study in healthy subjects was that within the two groups as little as 21% to as much as 49% of the variability in s` was explained by SExc. This observation may be at least partly explained by the heterogeneity in shape of the systolic Doppler TDI signal, however, our findings suggest that it is also due in part to natural variability of resting heart rate in conjunction with the velocity staircase effect. Adjusting for heart rate in the multivariate analyses led to significant improvements in the prediction of s` from SExc, although in the two groups no more than 52% of the variability in s` was explained by the combination of heart rate and SExc. That variability in both heart rate and height could have substantial influences on the absolute magnitude of s` was demonstrated using calculations based on the regression equations and using real life heart rates and heights.

There were no correlations of age with s` or indeed, with any of the measures of LV long axis systolic motion in Group 1 in our study. By contrast, there were inverse correlations of age with both s` and SExc in Group 2. That there was no independent effect of age on s` in Group 2 after including SExc in the models indicates that the mechanism underlying the reduction in s` with age in that group was a decrease in the amplitude of mitral annular excursion. The relationship of aging with mitral annular or basal LV s` has been investigated in a number of previous studies and there is evidence for [24,27–31] and against [32,34,35,73] a decrease in s` with older age. A possible contribution to the divergence of findings in previous studies of age and LV s` is the variation in age ranges of the subjects in the different studies. Indeed, close inspection of the scatter plots of age and s` from the studies of Nikitin et al, Chahal et al and Dalen et al shows data which could also be consistent with relative preservation of s` up to an age of ~50 years, but with a decrease in s` beginning after this age [28,29,31]. Such a threshold age for an effect of age on s` is also consistent with our finding of the lack of correlation of age with s` in subjects < 50 years (Group 1) but an inverse correlation of age with s` observed in Group 2 with an age range of 40–80 years. Further investigation regarding the relationship of LV s` with age could be performed by reanalysis of data from larger published studies to see if it was consistent with the presence of an age threshold.

Studies in isolated myocardium show an acceleration of relaxation with increased frequency of contraction [74], and a shortening of the time constant of relaxation with pacing mediated increases in heart rate has been observed in both conscious dogs [75] and humans [48]. In the absence of any change in left atrial pressure also affecting the crossover point of the LV and left atrial pressures, by accelerating the drop in LV pressure a higher heart rate might therefore be expected to lead to a shorter isovolumic relaxation time. In the present study we measured the time interval between the end of long axis systolic motion and the commencement of early diastolic motion as the long axis motion equivalent of the isovolumic relaxation time (IVRT`). An inverse correlation between the IVRT` and heart rate was evident for both the septal and lateral LV walls and in both groups, and these findings are therefore consistent with an acceleration of LV relaxation in association with a higher heart rate. In contrast with the positive relationship we observed between heart rate and s` and despite the inverse relationship we observed between heart rate and IVRT`, heart rate was not related to septal or lateral e` or EDExc in either study group. There has only been limited previous study of the effects of heart rate on e` or its experimental equivalent of maximum lengthening velocity. In a papillary muscle study where frequency of contraction was varied, there was no change in the maximum lengthening velocity if the extent of shortening was unchanged [76]. Two pacing studies have been performed in intact animals but the findings were not conclusive. Nagueh et al performed right atrial pacing on anaesthetised dogs at rates of 109 and 133/min and found a decrease in both septal and lateral e` at the higher pacing rate [49]. However, other changes which may have influenced the e` were noted concomitantly with the increase in heart rate, including a reduction in LV end-diastolic pressure and volume. Caillet et al assessed the effect of heart rate in conscious dogs by measuring maximum lengthening velocity using ultrasonic crystals and found no effect of short-term atrial pacing at a rate of 140/min on MLV after matching for the extent of systolic shortening [77]. In the single human study of which we are aware, Burns et al investigated the effects of increasing heart rate on LV e` in a group of subjects with a dual chamber pacemaker [50]. A decrease in e` was observed during an increase in atrial rate from 67 to 80/min, however, the significance of this finding for the normal heart is uncertain as a number of the subjects were elderly, half of the subjects had a reduced LVEF and the effect of the increased heart rate on the extent of SExc or EDExc was not determined.

An important limitation of our study is that it was observational, therefore preventing any definite conclusions regarding causality. Nevertheless, the finding of inverse relationships of heart rate with the duration of long axis contraction was expected based on previous experimental and observational studies, and the main findings were similar in two complementary groups. There were some small differences between the septal and lateral LV walls, and whether these represent genuine differences or the play of chance cannot be determined by a single study. However, there is some evidence for the former given that differences between the behaviour of these walls have also been observed in other studies [30,31,43,78] and there can be differences in the shape of the septal and lateral systolic signals. In addition, our findings are based on normal variability in resting heart rates <100/min and thus they may not reflect the effects of either spontaneous or externally stimulated tachycardia on long axis systolic excursion or peak velocities. Yet, while superficially attractive, a study of long axis ventricular function in which heart rate is increased by atrial pacing in healthy human subjects would be difficult to interpret due to the reductions in stroke volume and LV end-diastolic size which inevitably accompany a pacing mediated increase in heart rate in the normal heart [49,61,65,66]. Lastly, an assumption was made for the analysis of this study that heart rate and anthropometric measures were linearly related to TDI variables, but there are reasons to also consider the possibility of allometric relationships [79]. However, the appearance of the graphs and plots of the residuals for the correlations of heart rate with SDur and s` were consistent with linear relationships, height is dimensionally appropriate for SExc and s` and thus did not require adjustment on theoretical grounds, and use of the more dimensionally appropriate BSA^0.5^ rather than BSA did not improve the correlation with any of the TDI variables shown in Table 4 (results not shown).

In conclusion, in adult subjects with a normal LVEF, resting heart rate is an inverse and independent correlate of LV long axis SDur and a positive and independent correlate of LV long axis s`. These observations regarding heart rate are consistent with previous descriptions in humans of the “velocity staircase” effect, which is directly related to the force-frequency relationship [59,60,62]. In contrast, and despite the finding of an inverse correlation of resting heart rate with IVRT`, implying acceleration of the LV pressure drop with increasing heart rate, there were no correlations of heart rate with either e` or EDExc. That there was only a moderate correlation between s` and SExc, in conjunction with the finding that both heart rate and height contribute to the prediction of s` from SExc, suggests that s` and SExc cannot be used interchangeably for the assessment of long axis systolic function. This has important implications for the interpretation of s` in both experimental or clinical studies as it suggests that adjustment for heart rate and height may be indicated, whereas it has not been uncommon to use s` in isolation to reflect LV long axis systolic function [80,81]. However, adjustment for heart rate in pathological states is even more complex as experimental studies have demonstrated that there can be an inverse effect of heart rate on contraction when myocardial function is abnormal [82–85]. While height provides a simple additional method of adjustment for s` with respect to SExc, we have not identified a specific mechanism to explain why there is a positive relationship of height with s` (but not with SExc) in this study. Lastly, our findings provide a possible insight into previous divergent data with regard to age effects on s`, suggesting that there may be preservation of s` and SExc till middle age, but then a subsequent deterioration in both.

## Supporting information

S1 File Excel file containing raw data. (XLSX)

## Abbreviations

BMI: body mass index
BP: blood pressure
BSA: body surface area
e`: peak velocity of early diastolic mitral annular motion
EDExc: early diastolic mitral annular excursion
IVRT`: difference in time between the end of tissue Doppler imaging long axis contraction signal and the beginning of early diastolic signal
LV: left ventricular
LVEDL: left ventricular end-diastolic length
LVEF: left ventricular ejection fraction
s`: peak velocity of systolic mitral annular motion
SDur: duration of long axis systolic signal
SExc: systolic mitral annular excursion
